# Analysis of Cervical Range of Motion in Subjects Affected by Temporomandibular Disorders: A Controlled Study

**DOI:** 10.3390/medicina60010037

**Published:** 2023-12-25

**Authors:** Alessandro Nota, Laura Pittari, Alessia Claudia Lannes, Chiara Vaghi, Clarissa Calugi Benvenuti, Simona Tecco

**Affiliations:** 1 Dental School, Vita-Salute San Raffaele University and Department of Dentistry, IRCCS San Raffaele Hospital, 20132 Milan, Italy; laura_pittari@hotmail.it (L.P.); alessialannes@gmail.com (A.C.L.); chiara.vaghi98@gmail.com (C.V.); c.calugibenvenuti@gmail.com (C.C.B.); tecco.simona@hsr.it (S.T.); 2 Department of Clinical Medicine, Public Health, Life and Environmental Sciences (MeSVA), University of L’Aquila, 24100 L’Aquila, Italy

**Keywords:** temporomandibular disorders, cervical range of motion, accelerometer, digital dentistry, craniomandibular disorders

## Abstract

*Background and Objectives*: The aim of this study were to compare the cervical ROM data obtained from accelerometer exams between patients suffering from TMDs (study group) and healthy patients (control group). *Material and Methods*: A sample of 43 young adult subjects (23 control subjects and 20 TMD patients) were included in this study and analyzed with the accelerometer (Baiobit™, BTS, Garbagnate Milanese, Milan, Italy) to assess cervical ROM using a standardized protocol, including the following occlusal conditions: mandibular rest position, clenching, clenching with cotton rolls, maximal intercuspation, and mandibular position with cotton rolls. The cervical ROM was measured in degrees and expressed as the mean and standard deviation. *Results*: TMD patients showed a reduced cervical extension compared to control subjects in all the conditions, with statistically significant relevance. Regarding the other movements, the differences were not statistically or clinically significant. *Conclusions*: Based on the results of the present study, it can be observed that temporomandibular disorders are associated with a decreased cervical extension range, while the remaining components of ROM do not seem to be significantly associated. The use of accelerometers in ROM analysis could be helpful in improving interdisciplinary communication between dentists and physiotherapists.

## 1. Introduction

Temporomandibular disorders (TMDs) consist of a group of pathologies affecting the masticatory muscles, the temporomandibular joint, and related structures [1].

These disorders can cause discomfort and pain in the jaw area, impacting the normal opening and closing of the mouth [2]. They can be broadly categorized into two main types: muscle disorders and joint disorders. Muscle disorders primarily involve the muscles controlling jaw movements, leading to symptoms such as muscle pain, stiffness, and fatigue. Causes may be linked to factors like stress, teeth clenching (bruxism), or muscle tension. On the other hand, joint disorders mainly involve the temporomandibular joints themselves, resulting in symptoms such as joint pain, clicking during jaw movement, and limitations in movement. Causes may stem from structural issues or dysfunctions in the joints. Often, temporomandibular disorders present a combination of muscle and joint symptoms [3].

Risk factors include stress, arthritis, dental problems, poor posture, excessive teeth clenching, and jaw injuries [4].

TMDs pose a substantial public health concern, impacting around 5 to 12% of the general population [5].

A study by Iodice et al. investigated the incidence of Temporomandibular Disorders (TMDs) in Italian adults. This study revealed that temporomandibular joint (TMJ) clicking is the most frequent symptom (30.7%), followed by TMDs-induced pain (16.3%) and TMJ crepitus (10.3%). The results suggest that TMDs is a common ailment among the Italian adult population. Gender, oral behaviors, and a positive history of previous facial trauma were significantly associated with TMDs-related pain and TMJ clicking. TMDs-related pain is more prevalent in females and less widespread than TMJ clicking. The analysis of the general Italian population found that TMDs-related pain is more common in women, while TMJ clicking is more widespread. Furthermore, it is noteworthy that both pains associated with TMDs and clicking of the TMJ joint could be attributed to preceding facial trauma and oral habits. Prevalence of temporomandibular disorder pain, jaw noises, and oral behaviors in an adult Italian population sample [6].

According to the results of Obamiyi et al., it has also been noted that these findings are not easily generalizable to the global population, as there are radiographic differences in craniofacial structures and the temporomandibular joint (TMJ) that cannot be overlooked among racial groups. For example, Chinese patients presented with more radiographic features suggestive of TMDs, whereas Indians showed the least, compared with subjects from the White, Black, and Hispanic racial groups [7].

The etiologies of TMDs are multi-factorial and can be attributed to both physical and psychosocial factors. Various predisponding factors have been identified, including genetic, hormonal, functional, and anatomical factors [8,9].

The connections between the cervical spine and the stomatognathic apparatus involve neurological, muscular, and postural aspects. Nerves innervating the cervical region are interconnected with those serving the stomatognathic apparatus. For instance, the trigeminal nerve, responsible for sensory innervation of the face and jaw, may have interconnections with cervical nerves, contributing to neurological communication between the two regions [10,11,12].

Many muscles are involved in both the mobility of the cervical spine and the movement of the stomatognathic apparatus. For example, certain neck muscles, such as the sternocleidomastoid, are also engaged in chewing and movements of the jaw. This muscular overlap can influence posture and coordinated movements between the head and the mandible [13,14,15].

The posture of the cervical spine can influence the balance and posture of the stomatognathic apparatus. Alterations in cervical posture can have downstream effects, influencing the position of the mandible, the closure of dental arches, and other aspects of the temporomandibular joint (TMJ) [15,16,17]. 

Pain originating from the musculoskeletal structures is magnified by chewing or other jaw movements or due to movements of the head or incorrect position [18,19,20]. Walczyńska-Dragon K et al. (2014) have investigated the presence of a statistically significant correlation between the posture of the head and TMD [21].

Literature also proved the possibility of coexistence, in the same patients, of cervical spinal pain (CSD) and TMD. From a clinical point of view, it has been observed that TMD patients suffer from CSD more frequently than healthy subjects [22,23,24,25].

Commonly, patients who experience pain during mandibular or chewing movements often look for a TMD evaluation with a dental specialist.

During the gnathological examination, the dentist often evaluates the cervical spine, for example, through palpation of the cervical and sternocleidomastoid muscles or electromyographic evaluation of the neck muscles. This is because there is evidence of neurological, anatomical, and functional connections between the cervical area of the spine and the stomatognathic apparatus [26,27,28].

The gnathological examination is a specialized clinical assessment aimed at evaluating masticatory function, the temporomandibular joint (TMJ), and surrounding structures. It begins with medical and dental history data collection, including symptoms such as jaw pain, headaches, and chewing problems. Subsequently, a comprehensive clinical examination of the head and neck is performed, with particular attention to the jaw region and TMJ. The examination includes an assessment of the patient’s posture, jaw mobility, and possible muscle tensions or asymmetries. This is followed by a detailed examination of the mouth, teeth, and gums to identify any dental problems or malocclusions. The relationship between mouth closure, dental occlusion, and jaw movement is carefully evaluated. The patient may be involved in specific movements to assess masticatory function and identify any abnormalities. In some cases, instrumental examinations such as X-rays or magnetic resonance imaging may be required for a more detailed view of the joints and surrounding structures. The assessment of the patient’s body posture is an integral part of the visit, as postural alterations can affect jaw function. Muscle tension and the presence of trigger points in the involved muscles are examined. In specific situations, it may be necessary to take dental impressions or models for a more detailed assessment of occlusion.

For the dentist, the functional evaluation of the muscles of the cervical spine (cervical sternocleidomastoid muscles and upper trapezium muscles) cannot be clinically easy [1].

Instrumental examinations are therefore useful to allow an assessment to be as objective as possible. More recently, accelerometers have been introduced in this field, allowing an objective evaluation of the amplitude of the main cervical movements (rotation, flexion-extension, and inclination). 

The ROM (Range of Motion) of the cervical spine is a common parameter in evaluating cervical disorders [29]. In clinical gnathology, the examination can be used to check for possible changes in cervical movements during the different functional conditions of the stomatognathic apparatus [30].

In addition, it can be an excellent tool to improve the interdisciplinarity between the TMD specialist and the physiatrist.

The evaluation of cervical spine mobility is a topic of high interest due to the determining role that this structure plays in the relationship between the head and the human body [31,32]. It was previously carried out by clinical evaluation, while successively specific parameters and evaluation methods were gradually developed, which also included instrumental analyses [33,34].

In particular, the cervical ROM stands out as a fundamental analysis in the evaluation of this district and consists of the active or passive measurement of the amplitude of three cervical movements: rotation, lateral inclination, and flexion-extension of the head [15,35,36]. The record is followed by a comparison of the obtained results with the healthy population average values or an analysis of the asymmetries between the two body sides. 

In fact, the scientific literature suggests that cervical ROM alterations are related to various cervical pathologies [37]. A simple instrument, such as an accelerometer, can be used by the clinician to evaluate the ROM of the cervical spine. The accelerometer is an instrument able to detect and measure accelerations by calculating the force per unit of mass according to the second principle of dynamics (also called Newton’s second principle). Most accelerometers rely on the inertia of a mass contained within them when subjected to acceleration. An elastic element is responsible for the suspension of the mass, and various types of principles can be used to measure its displacement with respect to the structure of the device. This displacement will be clearly proportional to the intensity of the acceleration to which the mass is subjected. An accelerometer capable of detecting the displacement of its internal mass on the three axes of space (the three-axis accelerometer), thanks to the constant action on it by the force of gravity, becomes a tool able to detect not only the accelerations but also the inclination of a body, that is, the so-called “tilt sensing”.

The combination of an accelerometer with two other types of sensors—a gyroscope and a magnetometer (or digital compass)—represents a so-called IMU (Inertial Measurement Unit) or 9-axis accelerometer; a device capable of tracing any aspect related to the movement of a body. This technology is nowadays applied in several fields, including the medical one [38]. 

Usually, digital accelerometers for medical purposes include a wearable motion sensor that provides unrestricted movement and belts of various lengths to fit different areas of the body. The wireless sensor incorporates MEMS (Micro Electro-Mechanical Systems) inertial platforms, each consisting of a triaxial accelerometer, a triaxial gyroscope, and a triaxial magnetometer. In this text, the term "accelerometer" will be used, although all three components are fundamental to the analysis conducted. Sensor Fusion’s algorithms combine information from the gyroscope, accelerometer, and magnetometer and transmit it to the software via a Bluetooth connection. The sensor acquires and transmits the data to the PC for processing and automatic report generation. The software uses the sensor data to calculate amplitude angles for each movement within the cervical range of motion [39].

The accelerometer can also allow the evaluation of the range and speed of motion of the main movements of the head. Although it is a rather easy instrument to use, the correct execution of the examination requires the clinician to pay attention to ensuring that the movements of the head are performed correctly, without undesired movements of the shoulders.

In TMD clinical practice, this examination can be used to analyze variations in cervical movements between different functional conditions of the stomatognathic apparatus and to improve communication and interdisciplinarity between TMD specialists and physiatrists.

In accordance with a previous study, the accelerometer can be considered a reliable tool when a standardized protocol is applied [39].

It was hypothesized that there could be an influence of TMDs on the cervical ROM of the subjects affected. Therefore, the aim of this study were to compare the cervical ROM data obtained from accelerometer exams between patients suffering from TMDs and healthy patients.

## 2. Materials and Methods

A total of 43 subjects (27 F, 16 M; mean age 26.93 ± 7.58 years) were enrolled in this study between January 2023 and October 2023 and analyzed at the Department of Dentistry of IRCCS San Raffaele Hospital in Milan (Italy). The sample size was estimated a priori using the software G*Power (Heinrich Heine Universitat, Dusserldorf, Germany) on the basis of a pilot study performed on the first 10 subjects per group enrolled and analyzed in the present study. The preliminary data were used to calculate the effect size for the primary outcome (EXT) and estimate the minimum sample. Applying the lowest effect size obtained for the primary outcome among the different conditions (0.88) to the a-priori sample size analysis, a minimum number of 17 subjects for each group was required to achieve an 80% power with a 0.05 significance threshold.

This study was approved by the ethics committee of IRCCS San Raffaele Hospital (Milan, Italy) with the document “parere09/int/2023” 25 January 2023.

Subsequently, the subjects underwent an anamnestic and clinical examination in order to assess mandibular parameters according to DC/TMD criteria.

All participants were over 18. They were required to meet the following criteria: absence of spontaneous pain in the cervical spine; no systemic diseases that impacted cervical spine mobility (such as scoliosis, fractures, trauma, morphological alterations, and degenerative diseases); no craniosacral trauma; no craniosacral physiotherapy treatment; or orthodontic and dental treatment. Furthermore, the subjects were included in this study group when affected by myalgia, local myalgia, myofascial pain with spreading, and myofascial pain with referral, while they were included in the control group if not affected by TMDs.

Subjects were divided into two groups: a study group composed of 20 patients suffering from TMDs and a control group composed of 23 healthy patients free from TMDs. All patients underwent cervical ROM recordings and test sessions in five different mandibular conditions with the accelerometer (Baiobit™, BTS, Garbagnate Milanese, Milan, Italy) as performed in a previous study [39]:

T01 (REST): mandibular rest position

T02 (MAX. INT.): maximum intercuspation

T03 (COTTONS): mandibular rest position with cotton rolls

T04 (CLE.COT.): clenching on cotton rolls

T05 (CLENCHING): clenching in maximum intercuspation

**Figure 1:**
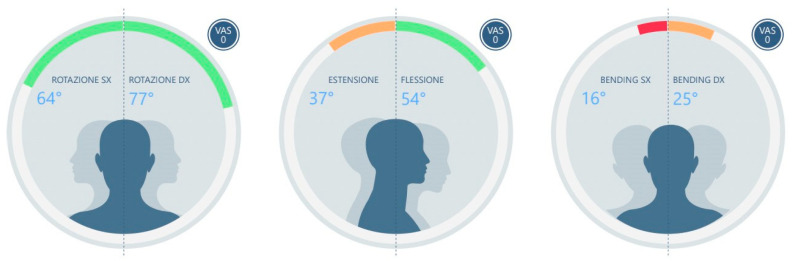
An example of the report of cervical ROM with the accelerometer Baiobit.

For each session, the following parameters were analyzed (Figure 1):

- ROT L (left rotation)
- ROT R (right rotation)
- ASIM ROT (absolute difference between right and left rotation angles)
- EXT (extension)
- FLEX (flexion)
- BEN L (left side bending)
- BEN R (right side bending)
- ASIM BEN (absolute difference between right and left lateral bending angles)

The exams were performed by an expert operator, who constantly checked the patient’s correct execution of the movements with no movements of the shoulders during the exam.

### Statistical Analyses

A complete data set were obtained and analyzed using descriptive statistics (mean and standard deviation). After confirming the normal distribution with the Kolmogorov–Smirnov test, inferential statistics with the student’s *t*-test for independent samples were applied to compare the means of the two groups. The threshold for the *p*-value was set at *p* < 0.05 using StatPlus (AnalystSoft Inc., Brandon, FL, USA) software. Standardized effect sizes with cohen’s d were calculated when statistical significance was observed.

## 3. Results

From the sample, descriptive and inferential statistics for each variable describing the different methods of analysis with different mandibular positions (REST, MAX. INT., COTTONS, CLE.COT., CLENCHING) have been calculated and reported in Table 1, with “Test” for TMD patients and “Control” for healthy ones. 

The difference in cervical extension movement (EXT) between the two groups was evaluated: in TMD subjects (study group), the EXT was significantly lower than in the control group. In this study group, EXT ranged from a minimum of 45.60 ± 13.90 degrees in REST to a maximum value of 49.55 ± 14.02 degrees in MAX.INT., while in control subjects it ranged from 61.70 ± 17.13 degrees in COTTONS to a maximum of 66.17 ± 14.76 degrees in REST.

In addition, a bending asymmetry was observed in both groups, with no significant differences between the two groups except in CLE.COT. In this condition, this study group showed a bending asymmetry of 6.60 ± 5.09 degrees, while the control group showed a bending asymmetry of 4.09 ± 4.04 degrees (*p* < 0.05). Therefore, the range of the asymmetry between right and left bending was similar in the two groups.

In particular, this study group showed a significantly lower bending amplitude on the right side compared to the control group in 4 out of 5 conditions, and it is on average always lower than on the left side in the same group. 

## 4. Discussion

The cervical system movement was previously carried out by clinical evaluation, while successively specific parameters and evaluation methods were gradually developed, which also included instrumental analyses [40,41]. 

Over the years, the application of appropriate instruments such as goniometers or inclinometers and specific devices for the analysis of cervical ROM became more popular, and in the last decades, the use of digital accelerometers was also introduced and partially analyzed by the previous literature [27,38,39,42,43,44]. 

In this study, the subjects underwent an anamnestic and clinical examination in order to assess mandibular parameters according to DC/TMD criteria investigating both muscular and articular disorders [4]. The amplitude of cervical ROM was assessed in TMD subjects using an accelerometer and compared to healthy subjects. 

Data showed a significantly reduced amplitude of cervical extension movements in TMD subjects of about 15 degrees with respect to healthy subjects in all the occlusal conditions.

In addition, subjects with TMDs showed reduced right bending compared to both the contralateral and control group patients, with a difference of about 4–5 degrees that could be considered of low clinical relevance. A mild asymmetry in lateral bending was detected for both this study and control groups; thus, the reduced right bending could be within the range of normal asymmetry.

From a clinical point of view, these results seem to suggest that the cervical extension movement is most affected by the presence of TMDs, so future studies could deeply investigate the relationship between the presence of TMDs and reductions in cervical extension range and, meanwhile, the effect of TMD therapies on this movement.

The restriction of extension movement in TMD patients, as assessed by ROM, is multifactorial and without a universal explanation. TMDs affect both the muscles involved in chewing and those of the neck, creating tension and spasms that impede movement. The resultant tension may lead to limited movement [13,14]. Additionally, by eliciting pain, TMDs may cause reflexive protection, which leads to neck movement restrictions intended to alleviate discomfort [45]. 

Patients with TMDs may develop postural compensations to ease pain associated with jaw movements. Such compensations may also be detected in neck posture [16]. 

Moreover, psychological concerns such as stress and anxiety, which are prevalent in TMD sufferers, can exacerbate general muscle tension [46,47]. 

This result agrees with a study by Grondin et al. (2015), who compared a sample of subjects affected by TMDs with a control group of healthy and asymptomatic subjects; rotation and flexion/extension movements were significantly reduced in individuals with TMDs, thus identifying an association between TMDs and a condition of reduced mobility of the cervical spine [44]. It could be hypothesized that Grondin et al. found an influence on rotation differently from the present study because they analyzed a sample of subjects with TMDs associated with headaches differently from the present sample. 

In addition, while the same research group observed in subjects with TMDs that the mandibular position appears to be able to affect cervical ROM [27], another study by Baldini et al. showed that the mandibular position does not seem to be able to influence cervical ROM in healthy subjects who are asymptomatic and free from TMDs [38]. 

A previous study by Nota et al. evaluated the extension in a sample of healthy subjects, observing extension values ranging from a minimum of 59.41 ± 16.10 degrees in the rest position with cottons to a maximum of 63.09 ± 15.16 degrees in the rest position [39]. These results look comparable to the ranges of the present study, which are (Table 1) between 61.70 ± 17.13 degrees (COTTONS) and 66.17 ± 14.76 degrees (REST). Thus, the observed ranges seem to be clinically reliable.

The association between a reduced extension and the presence of TMDs could be due to the close anatomical, biomechanical, and neurophysiological link between the masticatory system and the cervical structure. In fact, the scientific literature of the last 30 years has given attention to the presence of cervical pain in a lot of TMD patients [48,49].

From a clinical point of view, the assessment of the cervical ROM through the accelerometer resulted in an analysis that can be useful to improve the cooperation between the dentist and the physiotherapist in the management of clinical cases with TMDs. In this study, the test with an accelerometer examination was performed by an experienced dentist in the TMD field who was able to objectively record the cervical ROM, a parameter that is generally difficult to precisely assess clinically. Nowadays, the accelerometer provides an objective cervical ROM measurement, useful for TMD therapies, although poor repeatability was observed during the assessment of rotation and later bending asymmetry [39,40]. 

This study has some limits, as the sample size should be increased and the age of the participants limited to a range of young adults; another important limitation is not having identified various degrees of TMD severity and thus not being able to verify if the ROM values could change according to this. This will certainly be the aim of future studies with larger samples.

## 5. Conclusions

The present research seems to suggest that subjects with TMDs have an influence on cervical extension movement that results in reduced results, even in a sample of young adults. Further analysis on wider samples and with different age distributions is necessary to deeply investigate the other movements. This examination offers a fast and low-cost alternative to objectively quantifying ROM limitation in patients with TMDs and could be helpful in improving interdisciplinary communication between dentists and physiotherapists.

## Figures and Tables

**Table 1:** Results of descriptive statistics and comparison between the two groups (n = 43).

Task	Variable	Test Group (TMDs Patients)	Control Group (Healthy Patients)	*p*-Value
**T01** **(REST)**	ROT L	75.55 ± 11.36	75.19 ± 7.17	0.451
	ROT R	72.40 ± 11.29	74.52 ± 9.31	0.260
	ASIM ROT	6.95 ± 4.43	7.26 ± 5.47	0.421
	EXT	45.60 ± 13.90	66.17 ± 14.76	**<0.000** **Effect size 1.43**
	FLE	61.65 ± 11.25	58.30 ± 10.69	0.169
	BEN L	43.30 ± 7.44	40.43 ± 7.93	0.320
	BEN R	38.25 ± 7.98	43.13 ± 6.67	**0.021** **Effect size 0.66**
	ASIM BEN	7.05 ± 4.70	5.74 ± 4.44	0.183
**T02** **(MAX. INT.)**	ROT L	73.95 ± 10.84	75.39 ± 6.69	0.309
	ROT R	70.10 ± 10.14	73.43 ± 9.60	0.144
	ASIM ROT	6.95 ± 6.34	6.65 ± 5.98	0.439
	EXT	49.55 ± 14.02	64.17 ± 14.92	**0.001** **Effect size 1.00**
	FLE	60.35 ± 12.61	59.22 ± 9.21	0.374
	BEN L	42.65 ± 8.57	42.65 ± 7.11	0.500
	BEN R	38.50 ± 8.70	40.22 ± 9.06	0.270
	ASIM BEN	6.55 ± 4.99	6.78 ± 4.14	0.437
**T03** **(COTTONS)**	ROT L	75.40 ± 10.89	75.04 ± 8.60	0.455
	ROT R	70.35 ± 8.77	73.74 ± 8.32	0.107
	ASIM ROT	8.45 ± 8.12	7.04 ± 4.31	0.252
	EXT	47.45 ± 12.98	61.70 ± 17.13	**0.002** **Effect size 0.94**
	FLE	61.60 ± 9.09	58.04 ± 11.04	0.132
	BEN L	42.20 ± 7.90	42.17 ± 7.08	0.496
	BEN R	37.65 ± 7.95	42.04 ± 7.50	**0.039** **Effect size 0.57**
	ASIM BEN	6.65 ± 5.04	5.26 ± 4.00	0.170
**T04** **(CLE. COT.)**	ROT L	76.65 ± 8.84	75.35 ± 7.01	0.304
	ROT R	70.50 ± 9.29	74.96 ± 9.09	0.065
	ASIM ROT	7.65 ± 6.71	5.87 ± 4.12	0.162
	EXT	47.95 ± 12.24	62.96 ± 14.10	**<0.000** **Effect size 1.13**
	FLE	60.85 ± 8.45	62.93 ± 9.28	0.240
	BEN L	43.30 ± 6.75	42.61 ± 6.40	0.370
	BEN R	37.90 ± 7.96	42.00 ± 6.95	**0.045** **Effect size 0.55**
	ASIM BEN	6.60 ± 5.09	4.09 ± 4.04	**0.046** **Effect size 0.55**
**T05** **(CLENCHING)**	ROT L	73.45 ± 9.22	75.48 ± 8.48	0.235
	ROT R	67.85 ± 9.32	74.96 ± 10.84	**0.015** **Effect size 0.70**
	ASIM ROT	7.30 ± 6.44	10.17 ± 7.67	0.100
	EXT	49.40 ± 12.65	64.83 ± 15.09	**<0.000** **Effect size 1.11**
	FLE	56.90 ± 13.60	62.22 ± 12.67	0.103
	BEN L	40.10 ± 8.44	41.91 ± 7.09	0.232
	BEN R	36.85 ± 7.81	41.57 ± 7.20	**0.026** **Effect size 0.63**
	ASIM BEN	6.25 ± 4.70	4.43 ± 2.55	0.072

## Data Availability

Individual patients’ data are not shown for privacy reasons but is available upon reasonable request at Vita-Salute San Raffaele University in Milan, Italy.
